# The Effects of Adding Di-Tan Decoction (DTD) and/or Electroacupuncture (EA) to Standard Swallowing Rehabilitation Training (SRT) for Improving Poststroke Dysphagia (PSD): A Pilot, Single-Centred, Randomized Trial

**DOI:** 10.1155/2022/2011597

**Published:** 2022-12-08

**Authors:** Xiangzhi Shao, Bing Chen, Lielie Zhu, Lili Zhu, Jinyihui Zheng, Xinyu Pu, Jiajun Chen, Jianning Xia, Xinming Wu, Jiacheng Zhang, Dengchong Wu

**Affiliations:** Department of Rehabilitation, Wenzhou Hospital of Traditional Chinese Medicine, Wenzhou 325000, China

## Abstract

**Objectives:**

To evaluate the effect of adding Di-tan decoction (DTD) and/or electroacupuncture (EA) to standard swallowing rehabilitation training (SRT) on improving PSD.

**Methods:**

In total, 80 PSD patients were enrolled and randomly assigned to the DTD, EA, DTD + EA or control group at a 1 : 1 : 1 : 1 ratio. All patients received basic treatment and standard SRT. The DTD group received DTD orally, the EA group received EA, the DTD + EA group received both DTD and EA simultaneously, and the control group received only basic treatment and standard SRT. The interventions lasted for 4 weeks. The outcome measurements included the Standardized Swallowing Assessment (SSA) and Swallowing-Quality of Life (SWAL-QOL), performed and scored from baseline to 2, 4, and 6 weeks after intervention, and the Videofluoroscopic Dysphagia Scale (VDS), scored at baseline and 4 weeks after intervention. Scores were compared over time by repeated-measures analysis of variance (ANOVA) among all groups. Interactions between interventions were explored using factorial design analysis.

**Results:**

(1) The effective rates (ERs) for PSD treatment were higher in the DTD, EA and DTD + EA groups than in the control group (all *P* < 0.05). The ER was higher in the DTD + EA group than in the DTD or EA group (both *P* < 0.05). (2) There were significant group effects, time effects and interactions for the SSA and SWAL-QOL scores (all *P* < 0.05). All groups showed decreasing trends in SSA scores and increasing trends in SWAL-QOL scores over time from baseline to 6 weeks after intervention (all *P* < 0.01). (3) Factorial design analysis for ΔVDS showed that there was a significant main effect for DTD intervention (*F* = 11.877, *P* < 0.01) and for EA intervention (*F* = 29.357, *P* < 0.01). However, there was no significant interaction effect between DTD and EA (*F* = 0.133, *P* = 0.717). Multiple comparisons showed that the DTD, EA and DTD + EA groups all had higher ΔVDS values than the control group (*P* < 0.05). The DTD + EA group had a higher ΔVDS than the DTD or EA group (both *P* < 0.05). (4) Most adverse reactions were mild and transient.

**Conclusions:**

Adding DTD or EA to SRT can better improve PSD than applying SRT alone. Adding DTD and EA simultaneously can accelerate and amplify the recovery of swallowing function versus DTD or EA alone, and both are effective and safe treatments, alone or jointly, for PSD and are a powerful supplement to routine treatments.

## 1. Introduction

Dysphagia symptoms are present in approximately 37%–78% of patients after acute stroke [1]. Nearly 75% of patients can recover to an oral diet at discharge, but 11%–13% of them still suffer from sequelae with dysphagia even after six months [2]. Poststroke dysphagia (PSD) severely affects the quality of life (QOL) of patients by causing aspiration pneumonia, malnutrition, dehydration, and depression, leading to a poor prognosis or even death [3]. There is enough evidence to show that early and effective rehabilitation treatments for PSD are essential to alleviating symptoms, shortening the course of the disease, and improving prognosis [4].

At present, there are quite a few rehabilitation treatments applied for PSD, such as swallowing rehabilitation training (SRT), compensation therapy, physiotherapy, and alternative therapy [4–6]. However, most treatments have only a temporary and relatively limited effectiveness against PSD, making PSD a refractory disease. Further exploration of comprehensive rehabilitation therapy for PSD under multidisciplinary management is needed.

Traditional Chinese medicine (TCM), including Chinese medicinal decoctions and acupuncture therapy, has been widely used in the management of a many diseases in East Asia and has been reported in many studies [7–10]. According to TCM theory, PSD is related mainly to meridian obstruction or phlegm dampness [11]. As a classic Chinese medicine formula, “Di-tan decoction” (DTD), which was developed by a famous TCM doctor, Dong Su, in 1449 with the specific function of “resolving phlegm to open the orifices” [12], has been prescribed to patients with neurological disorders [10] and has achieved a certain effectiveness for stroke in clinical practice [13–16]. As a form of complementary and alternative medicine, the therapeutic effects of acupuncture are mediated by inserting a needle at specific acupoints on the body surface to deliver stimulation via manual rotation or electric pulses [17, 18]. Some randomized controlled trials (RCTs) have shown that acupuncture may alleviate symptoms of dysphagia [19–21]. In particular, it is worth mentioning that electroacupuncture (EA), as an extension technique of acupuncture based on traditional acupuncture combined with modern electrotherapy, has been regarded as a promising method to treat PSD [22, 23].

However, there remains a lack of sufficient evidence to recommend the routine use of EA for dysphagia after stroke [22, 24]. Most reports on the application of Chinese medicinal decoctions for the treatment of neurological disorders involve small samples and restricted designs and are low-quality studies [10, 25]. In addition, in Chinese clinical practice, using a decoction combined with EA was believed to have a certain synergistic effect on the improvement of dysphagia [13, 25], but there is still a lack of sufficient evidence to support the conclusions at present.

Thus, we designed a randomized controlled trial to determine the individual effects of DTD and EA on the improvement of swallowing function in patients with ischaemic stroke and the interactive effect on PSD. We hypothesized that adding either DTD or EA to standard SRT could better improve PSD and that the intervention effect of DTD and EA could be synergistic.

## 2. Materials and Methods

### 2.1. Study Design

**Figure 1:**
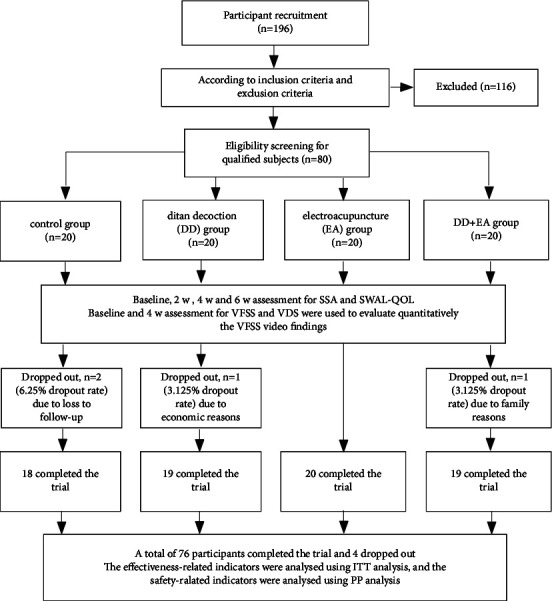
Flowchart for this study. Note: DTD: di-tan decoction; EA: electroacupuncture; SSA: standardized swallowing assessment; SWAL-QOL: swallowing-related quality of life; VFSS: video fluoroscopic swallowing study; VDS: videofluoroscopic dysphagia scale; ITT: intention-to-treat; PP: per-protocol.

The study was a randomized, single-blind clinical trial and was conducted in the Department of Rehabilitation, Zhejiang Chinese Medical University Affiliated Wenzhou Hospital of Traditional Chinese Medicine. A total of 196 patients who developed PSD and were admitted to the rehabilitation department in our hospital from June 2021 to June 2022 were enrolled. A total of 80 participants, including 60 males and 20 females, were enrolled according to the inclusion criteria and exclusion criteria listed below, and all of them were of Han ethnicity. This trial was approved by the Ethics Committee of Zhejiang Chinese Medical University Affiliated Wenzhou Hospital of Traditional Chinese Medicine (No. WTCM-KT-2021059), and written informed consent was obtained from all participants in accordance with the Declaration of Helsinki. The study workflow is shown in Figure 1.

### 2.2. Inclusion Criteria

(1) age between 50 and 85 years; (2) meeting the diagnostic criteria for ischaemic stroke, listed in the Diagnostic Criteria of Cerebrovascular Disease in China (version 2019) [26]; (3) a primary diagnosis of stroke between 14 and 60 days since onset confirmed by magnetic resonance imaging or computerized tomography scans; (4) meeting the diagnostic criteria for dysphagia, based on Scottish Intercollegiate Guidelines Network 2010 [27]; and (5) normal cognitive function and hearing for good compliance with examination and treatment.

### 2.3. Exclusion Criteria

(1) swallowing disorders caused by conditions other than stroke; (2) local throat lesions; (3) thyroid disease, local skin infection or ulcer; (4) severe heart, lung, liver, kidney disease, or unstable vital signs; (5) previous idiopathic epilepsy or epilepsy caused by other neurological disorders; or (6) metal implants in the body, such as pacemakers, cochlear implants and neck vascular stents.

### 2.4. Intervention

#### 2.4.1. Control Group

The patients in the control group received only basic treatment and SRT. According to the Guidelines of Diagnosis and Treatment of Acute Ischaemic Stroke in China (2018) [28], the basic treatment was the necessary supportive treatment for patients with ischaemic stroke, including antiplatelet, lipid-lowering, blood pressure control, and blood glucose control medications. Standard SRT included active or passive exercise of the oral, facial, and lingual muscles, sensory stimuli, and some specialized methods, such as the Mendelsohn manoeuvre, supraglottic and superglottic manoeuvres, and swallowing efforts.

#### 2.4.2. Di-Tan Decoction (DTD) Group

The patients in the DTD group were treated with DTD on the basis of basic treatment and SRT, similar to those in the control group. The DTD was given orally by intermittent oro-oesophageal tube (IOET) feeding with the following prescription: *Arisaema erubescens* Schott. 15 g, *Acorus tatarinowii* Schott. 15 g, *Poria cocos* Wolf. 15 g, *Citrus aurantium L*. 10 g, *Pinellia ternata* Breit. 6 g, *Citrus reticulata* Blanco. 6 g, *Panax ginseng* C. A. Mey. 20 g, *Glycyrrhiza uralensis* Fisch. 9 g, *Bambusa tuldoides* Munro. 12 g. All Chinese medicines used were standard decoction 200 ml, which was boiled by the staff of the hospital decocting room. The method was as follows: 1 dose a day, twice orally in the morning and evening, for 4 weeks.

#### 2.4.3. Electroacupuncture (EA) Group

**Figure 2:**
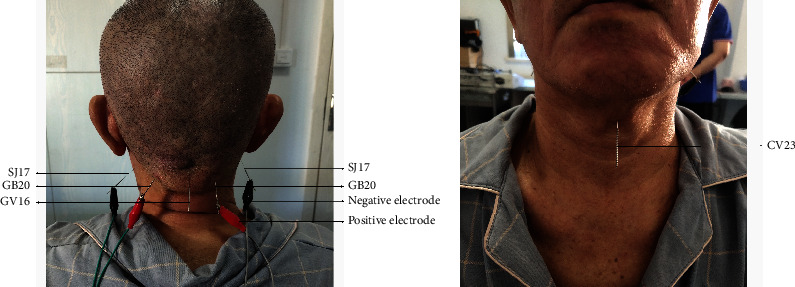
SJ17, GB20, and GV16 acupoints. (a) Back. (b) Front.

The patients in the EA group were treated with EA on the basis of basic treatment and SRT, similar to those in the control group. EA treatment was given as follows: The target acupoints were Linquan (CV23), FengChi (GB20), Yifeng (SJ17) and Fengfu (GV16). The EA operation method added modern electrotherapy to manual acupuncture as follows: The lian quan (CV23) was needled oblique towards the root of the tongue at a depth of 0.5–1.0 cun (1 cun = 33.3 mm); Fengchi (GB20) was needled towards the nasal apex at a depth of 0.5–1.2 cun. Yifeng (SJ17) was needled perpendicularly with a depth of 0.5–1 cun. Fengfu (GV 16) was needled perpendicularly with a depth of 0.5–0.8 cun. After the “de qi” sensation was felt by the acupuncturist, the needles needled in the acupoints mentioned above were connected to an acupoint stimulator (KWD-808I, Multi-PURPOSE HEAL TH DEVICE, Yingdi Electronic Medical Device Co., Ltd., Changzhou, China) using electrodes, with a continuous wave of frequency of 2 Hz for 30 min, and the intensity of EA was set according to the maximum tolerated intensity of each subject (between 0.9 mA and 3 mA) [23]. Each needle was kept once daily for 30 min for 4 weeks consecutively. All EA treatments were performed by an acupuncturist. The SJ17, GB20, and GV16 acupoints are shown in Figure 2.

#### 2.4.4. DTD Combined with EA (DTD + EA) Group

The patients in the DTD + EA group received DTD and EA treatments simultaneously, as well as basic treatment and SRT. The operations of the interventions and the entire course of treatment were all similar to those in the other three groups.

### 2.5. Baseline Characteristics Evaluation

The characteristics of demographic, clinical and medical history variables of all subjects, including age, sex, stroke course, National Institute of Health Stroke Scale (NIHSS) [29], comorbidity, age-adjusted Charlson comorbidity index (aCCI) [30] and the distribution of lesion location among patients, were recorded.

### 2.6. Outcome Measurement

#### 2.6.1. Standardized Swallowing Assessment (SSA)

The SSA consisted of three steps. The first was the clinical examination, including conscious level, control of the head and trunk, breathing, closure of the lip, soft palate movement, laryngeal function, pharyngeal reflex, and autonomic cough. Then, in Stage 1, 5 mL of water was given to the patient three times. In Stage 2, if swallowing was normal in Stage 1, 60 mL of water was given to the patient. SSA scores ranged from 17 to 46, with a higher score indicating a decreased swallowing ability [31]. SSA was assessed and scored at baseline, 2 weeks, 4 weeks, and 6 weeks after intervention.

#### 2.6.2. Swallowing-Related Quality of Life (SWAL-QOL) Questionnaire

The SWAL-QOL questionnaire consisted of 44 items and was specifically designed to evaluate various aspects of the quality of life of patients including those related to the physiological, psychological, emotional and social communication domains, with a higher score indicating a higher quality of life (QOL) [32]. The SWAL-QOL questionnaire was administered and scored at baseline and 2 week, 4 weeks, and 6 weeks after the intervention.

#### 2.6.3. Video Fluoroscopic Swallowing Study (VFSS) and Videofluoroscopic Dysphagia Scale (VDS)

VFSS has traditionally been regarded as the “gold standard” for assessing swallowing function [33]. The operation process of VFSS was as follows: 200 mg barium was added to 286 mL water to make radiopaque material (60%); rice flour was added to make a thin liquid and a thick liquid, and cake was used as solid food. Each participant sat upright in the fluoroscopy chair with the head and neck in the neutral position and the chest and abdomen in the true lateral plane. Patients sequentially consumed the above liquids starting at 2 mL, subsequently increasing to 5 mL and then to 10 mL. Fluoroscopy was used to image swallowing in lateral and anteroposterior projections. The fluoroscopy images included the lips anteriorly to the pharyngeal wall posteriorly and the soft palate superiorly to the 6th cervical vertebra. These images were recorded at a speed of 30 frames per second and stored digitally in a computer for further analysis. The result of VFSS showed video segments that were difficult to quantify, so VDS were introduced to semiquantitatively score the findings of VFSS [34]. VDS scores ranged from 0 to 100, with a higher score indicating a lower swallowing ability [35]. All patients were assessed by VFSS and scored as VDS at baseline and 4 weeks after intervention.

#### 2.6.4. The Effective Rate Assessment

With the change in VDS (ΔVDS) scores between baseline and 4 weeks as a reference, the effective rate (ER) for the treatment of PSD was calculated using the following formula: (VDS scores at baseline−VDS scores at 4 weeks)/VDS scores at baseline ×100%. The efficacy was graded by ER quartile as follows: completely healed (>75%), markedly effective (50%–75%), effective (25%–50%), and ineffective (≤25%) [10].

### 2.7. Adverse Events

Adverse events or all unexpected responses were recorded by physicians. In this trial, the adverse events were represented as the cumulative number of specific events occurring during the implementation of the intervention. The incidence of adverse events was calculated as the cumulative number of adverse events/the total number of intervention events. In addition, all new aspiration pneumonia during the entire 6-week follow-up was recorded as a dysphagia-related complication.

### 2.8. Sample Size Calculation

Sample size calculations were performed to determine the number of participants needed to detect effect sizes. We estimated the needed sample size of all the outcome indicators mentioned above and found that the sample size required in the SSA indicator was largest among all indicators. Therefore, we selected the SSA scores as the main referent of the sample size calculation. The procedure was as follows: based on data from our previous trials, the respective mean and standard deviation (SD) of SSA scores were 25.8 ± 5.6 in the control group, 23.9 ± 4.8 in the DTD group, 21.5 ± 3.9 in the EA group and 19.5 ± 4.5 in the DTD + EA group at the end of the interventions. A sample-size calculation was conducted using Power Analysis and Sample Size System version 15 (PASS 15), with a type I error of 5% (*α* = 0.05) and 80% power (*β* = 0.2). An established sample size of 72 participants in this trial was enough to reveal significant differences between the arms. In total, a targeted sample size of 80 participants, which allowed for a 10% drop-out rate, was established.

### 2.9. Statistical Analysis

Statistical analysis was performed using IBM SPSS software Version 25. The effectiveness-related indicators were analysed on the intention-to-treat (ITT) basis, while the safety-related indicators were analysed on the per-protocol (PP) basis. For continuous data, normally distributed variables are expressed as the means ± SD, while skewed variables are expressed as the median (interquartile range, IQR). After normality of the data was checked using the Shapiro-Wilk (S-W) test and homogeneity of variance was checked using Levene's test, continuous data between groups were assessed using one-way analysis of variance (ANOVA). The continuous data consisting of repeated assessment were analysed by repeated-measures ANOVA, in which the Mauchly spherical test was performed first, and then the Greenhouse-Geisser method was used to correct if the data did not meet the spherical hypothesis. In addition, the potential interactions between interventions were explored using a factorial design. Categorical data were assessed using the chi-squared test or Fisher's exact test. All multiple comparisons were corrected with the Bonferroni method. The results were considered significant at a *P* value of <0.05, two-tailed.

## 3. Results

### 3.1. Baseline Characteristics of General Data for Subjects

In total, 76 subjects completed the study (19 in the DTD group, 20 in the EA group, 19 in the DTD + EA group and 18 in the control group), and 4 subjects dropped out (1 in the DTD group, 1 in the DTD + EA group and 2 in the control group) owing to family reasons or loss to follow-up before completing the study. The 4 who dropped out were included in the analysis using their baseline values. No significant differences were found in the median age, sex proportion, median course of stroke, comorbidity proportion, aCCI, NIHSS or the distribution of the lesion location among the four groups (all *P* > 0.05). The characteristics of the demographic, clinical and medical history variables of the study are summarized in Table 1.

### 3.2. The Effective Rate Evaluation after Intervention for PSD

With the change in VDS (ΔVDS) scores between baseline and 4 weeks as a reference, the effective rate (ER) for the treatment of PSD in the four groups is listed in Table 2. Among all 80 patients, the highest ER was 78.0%, and the mean ± SD of ER was 45.6 ± 18.0%. The ER of 4 who dropped out was 0.0% because this was evaluated with baseline data. All ERs in the DTD, EA and DTD + EA groups were higher than those in the control group (all *P* < 0.05). The ER in the DTD + EA group was higher than that in the DTD or EA group (both *P* < 0.05). According to the efficacy graded by ER quartile, there was a higher proportion of markedly effective patients distributed in the DTD + EA and EA groups (both *P* < 0.05).

### 3.3. Changes in SSA Scores after the Intervention over Time

**Figure 3:**
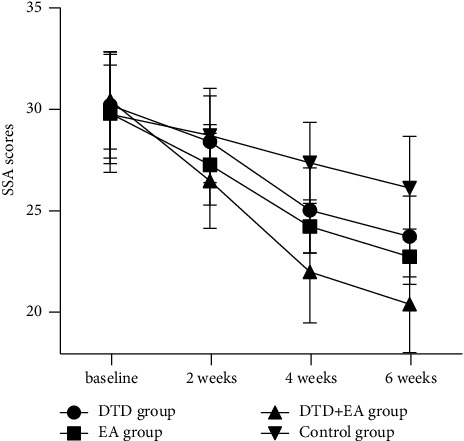
Trends over time of the SSA in the four groups.

According to the repeated-measures ANOVA for the SSA scores, Machly *W* = 0.156, with *P* < 0.01, which did not meet the spherical hypothesis. Therefore, the Greenhouse-Geisser method was used for correction. The S-W test showed that the data in every group had a normal distribution with all having a *P* > 0.05. Levene's test showed homogeneity of variance with *P* > 0.05. There were significant time effects, group effects and interactions of time and group (*F* = 312.129, 10.765, 12.819; all *P* < 0.01, respectively) for the SSA scores. Further simple effect analysis showed that the simple effects of time were all significant in every group (all *P* < 0.01). According to multiple comparisons between different timepoints when fixing the group factor, all groups showed decreased trends in SSA scores over time from baseline to 6 weeks after intervention (all *P* < 0.05). However, the simple effect of group was not significant at baseline (*F* = 0.327, *P* = 0.806), but all were significant at 2 weeks, 4 weeks and 6 weeks after intervention (*F* = 4.296, 23.781, 24.880; *P* = 0.007, <0.01, <0.01, respectively). Multiple comparisons between groups showed that the SSA scores of the DTD + EA group were significantly lower than those of the control group at 2 weeks after intervention (*P* < 0.05). At 4 weeks and 6 weeks after intervention, the SSA scores in the DTD, EA and DTD + EA groups were all lower than those in the control group (all *P* < 0.05). In addition, the DTD + EA group showed lower SSA scores than the DTD group from 2 weeks to 6 weeks and showed lower SSA scores than the EA group at 4 weeks and 6 weeks (all *P* < 0.05). There were no significant differences in the SSA scores between the DTD and EA groups at any timepoint (all *P* > 0.05). The results are shown in Table 3 and Figure 3.

### 3.4. Changes in SWAL-QOL Scores after Intervention over Time

**Figure 4:**
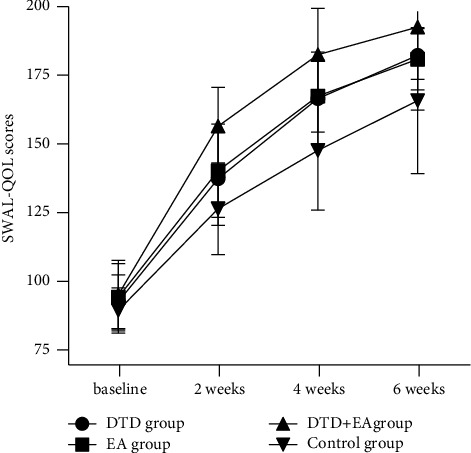
Trends over time of the SWAL-QOL in four groups.

According to the repeated-measures ANOVA for the SWAL-QOL scores, Machly *W* = 0.277, with *P* < 0.01, which did not meet the spherical hypothesis. Therefore, the Greenhouse-Geisser method was used for correction. The S-W test showed that the data in every group had a normal distribution with all having *P* > 0.05. Levene's test showed homogeneity of variance with *P* > 0.05. There were significant time effects, group effects and interactions of time and group (*F* = 741.809, 13.079, 3.741; *P* < 0.01, <0.01, <0.01, respectively) for the SWAL-QOL scores. Further simple effect analysis showed that the simple effects of time were all significant in every group (all *P* < 0.01). According to multiple comparisons between different timepoints when fixing the group factor, all groups showed increased trends in SWAL-QOL scores over time from baseline to 6 weeks after intervention (all *P* < 0.01). However, the simple effect of group was not significant at baseline (*F* = 1.055, *P* = 0.373), but all were significant at 2 weeks, 4 weeks and 6 weeks after intervention (*F* = 11.669, 13.367, 6.130; *P* < 0.01, <0.01, <0.01, respectively). Multiple comparisons between groups showed that the SWAL-QOL scores of the DTD + EA group were significantly higher than those of the control group at 2 weeks, 4 weeks and 6 weeks after intervention (all *P* < 0.05). At 4 weeks after intervention, the SWAL-QOL scores in both the DTD and EA groups were significantly higher than those in the control group (all *P* < 0.05). In addition, the DTD + EA group showed higher SWAL-QOL scores than the DTD or EA group at 2 weeks and 4 weeks (all *P* < 0.05). There were no significant differences in the SWAL-QOL scores between the DTD and EA groups at any timepoint (all *P* > 0.05). The results are shown in Table 4 and Figure 4.

### 3.5. The Decrease in VDS Scores between 4 Weeks and Baseline

**Figure 5:**
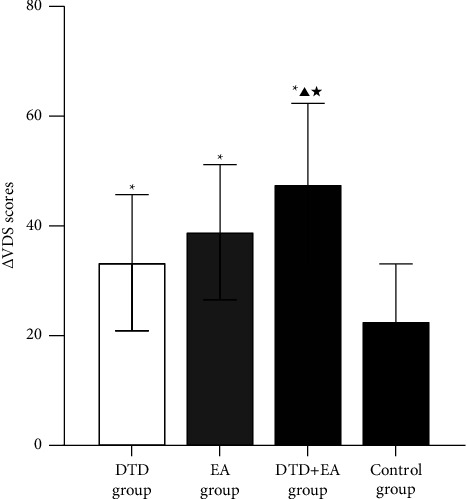
Comparisons of ΔVDS among the four groups. Note: ^*∗*^compared with the control group, *P* < 0.05; ▲compared with the DTD group, *P* < 0.05; ^★^compared with the EA group, *P* < 0.05.

The decrease in VDS (ΔVDS) was calculated as the difference in VDS scores between 4 weeks and baseline using the formula ΔVDS = VDS scores at baseline−VDS scores at 4 weeks. According to the 2 × 2 factorial design, one of the intervention factors in this trial had two options: giving Di-Tan decoction or not giving Di-Tan decoction. The other intervention factor also has two options: giving electroacupuncture or not giving electroacupuncture. Thus, this study consisted of the following 4 groups: (1) only Di-Tan decoction applied (DTD group); (2) only electroacupuncture applied (EA group); (3) Di-Tan decoction and electroacupuncture applied simultaneously (DTD + EA group); and (4) neither Di-Tan decoction nor electroacupuncture applied (control group). According to the S‒W W test, ΔVDS in each group followed a normal distribution (all *P* > 0.05). Levene's test showed homogeneity of variance with *P* > 0.05. A main effect of DTD intervention was observed (*F* = 11.877, *P* < 0.01), and a main effect of EA intervention was observed (*F* = 29.357, *P* < 0.01). However, there was no significant interaction effect of DTD and EA observed (*F* = 0.133, *P* = 0.717). Further multiple comparisons between groups showed that the DTD group, EA group and DTD + EA group all had significantly higher ΔVDS values than the control group (*P* < 0.05). The DTD + EA group had a significantly higher ΔVDS value than either the DTD group or the EA group (both *P* < 0.05), while there was no significant difference in ΔVDS scores between the DTD and EA groups (*P* > 0.05). The results are shown in Table 5 and Figure 5.

### 3.6. Adverse Events and Complications

The data of adverse events and complications were analysed on the per-protocol (PP) basis for the purpose of avoiding more conservative results. The adverse events in the DTD group included nausea or abdominal distension due to taking the decoction too quickly (3 times). The adverse events in the EA group included pain near the acupuncture site (4 times), mild bleeding at the acupuncture site (2 times), haematoma in acupoints after acupuncture (2 times) and transient discomfort related to tension (2 times). The adverse events in the DTD + EA group included pain in the acupuncture site (2 times), mild bleeding in the acupuncture site (2 times), haematoma in acupoints after acupuncture (2 times) and transient discomfort related to tension (2 times), nausea or abdominal distension due to taking too fast decoction (3 times). The incidence of adverse events based on the total number of intervention events in both the DTD + EA and EA groups was higher than that in the control group (both *P* < 0.05). In addition, the new aspiration pneumonia in the four groups during the entire 6-week follow-up was recorded. The incidence of new aspiration pneumonia based on the number of patients in both the DTD + EA and DTD groups was lower than that in the control group (both *P* < 0.05). The results are shown in Table 6.

## 4. Discussion

As two important traditional therapies, Chinese medicinal decoctions and acupuncture have been used in clinical practice for thousands of years, and both have shown particular effectiveness for many diseases even when facing powerful modern medicine [36].

Quite a few clinical studies have found that acupuncture (including electroacupuncture) has a certain effect on PSD [9]. Acupuncture stimulated local acupoints in the craniofacial and neck regions, which could prompt throat muscle contraction, regulate nerve function, and strengthen local blood circulation. At the same time, these acupoint regions are peripheral nerve branch distribution zones, such as the facial nerve, cervical transverse nerve, hypoglossal nerve, and mandibular nerve. Acupuncture to these regions could regulate autonomic nervous function and stimulate the re-establishment of reflexes related to swallowing [24]. Electroacupuncture using trace current close to human bioelectricity connects to the needle to enhance stimulus for peripheral receptors that regulate cerebral cortical excitability and improve dysphagia symptoms [22].

On the other hand, Chinese medicinal decoctions have another effect on the symptoms of neurological disorders, such as tongue stiffness, sudden falling and loss of consciousness, convulsions, hot temper, aphasia, insomnia, dryness of the pharynx, and cough with sputum, which are similar to poststroke symptoms [8, 10]. According to traditional Chinese medicine (TCM) theory, qi deficiency, blood stasis, and phlegm dampness are the basic factors of stroke [37]. Turbid phlegm is considered an important reason for swallowing disorders post stroke [38]. Theoretically, any decoction that can eliminate the “turbid phlegm” might improve the PSD.

According to TCM theory and clinical practice in China, we designed a study to compare the effectiveness of adding Di-Tan decoction and/or electroacupuncture to SRT for PSD in patients with a stroke course between 2 weeks and 6 months.

It is worth mentioning that we put the bottom line of the days from onset as 14 days for the sake of safety and to eliminate the influence of spontaneous recovery, owing to a previous study suggesting that 63.6% of PSD patients would recover spontaneously within 2 weeks [39].

First, based on balanced and comparable baseline data, we verified that there were more obvious improvements in the effective rate (ER) in the DTD and EA groups than in the control group, which indicates the positive intervention effectiveness of DTD and EA separately for swallowing function. Several meta-analyses summarized the RCTs about the effectiveness of EA or acupuncture for PSD, and the conclusions were quite consistent with our study [9, 22, 24]. However, there was a defect of low quality and high heterogeneity for most RCTs that used subjective clinical tools instead of the “gold standard” to evaluate swallow function and calculate the effective rate. In this trial, we used VFSS as the primary outcome measurement, which increased credibility. As a “gold standard” for evaluating swallow function, VFSS can dynamically observe the pathological changes of the mouth, pharynx, larynx and oesophagus in real time, which provides a relatively reliable and rapid basis for the qualitative diagnosis of swallowing disorders [33]. Considering that VFSS represented video segments and was difficult to quantify, we introduced VDS to semiquantitatively score the findings of VFSS [34, 40]. According to previous studies, the VDS is comprehensive and has high reliability and validity for evaluating swallow function in patients with PSD [40]. Therefore, compared with the other RCTs, implementing the VDS as a reference for calculating the effective rate of intervention in our study could be more objective and persuasive for drawing a conclusion. In addition, the DTD + EA group showed more significant improvement to the ER than the DTD or EA group, which put forwards preliminary clues for answering the question of whether combination therapy of DTD and EA has a more positive impact on PSD than DTD or EA alone.

Additionally, we used SSA to dynamically assess the change in swallowing function over time. SSA is a valuable screening tool that has demonstrated excellent sensitivity and good specificity for determining quick dysphagia and the risk of aspiration [31]. Then, the self-reported SWAL-QOL questionnaire was used to evaluate quality of life (QOL) with improvement in swallowing function [32].

During the whole 6-week follow-up, all four groups showed decreasing trends in SSA scores and increasing trends in SWAL-QOL scores compared with their baseline values (*P* < 0.05), which indicated that the patient's swallowing function gradually recovered with the implementation of the four intervention schemes (DTD, EA, DTD + EA, and SRT). Although the stroke course of the participants varied from 2 weeks to 60 days in this trial, positive rehabilitation efficacy still existed. At present, few studies have compared the change in swallowing function in patients with PSD over time under various intervention methods.

We further explored the data by fixing the factor of time, and multiple comparisons among groups showed that the SSA scores in either the DTD or EA group were significantly lower than those in the control group at 4 weeks and 6 weeks after intervention (all *P* < 0.05), which indicated that adding either DTD or EA intervention to SRT improved the swallowing function of patients with PSD more than the SRT intervention alone. Compared with that in the DTD or EA group, the improvement in SSA scores in the DTD + EA group over the control group emerged earlier, occurring at 2 weeks after intervention and lasting for the whole 6-week follow-up (all *P* < 0.05). The SSA scores in the DTD + EA group were lower than those in both the DTD and EA groups at 4 weeks and 6 weeks after intervention (all *P* < 0.05), which indicated that adding DTD and EA simultaneously to SRT could accelerate and amplify the recovery of swallowing function. For SWAL-QOL scores, conclusions from multiple comparisons among groups were similar to those for SSA scores, which indicated that there were corresponding improvements in swallowing-related QOL with the progression of swallowing function. In addition, we found that regardless of SSA or SWAL-QOL, there were no differences between the DTD and EA groups at any timepoint (all *P* > 0.05), which may hint that the two interventions were almost equal in their effect on PSD. However, at the 6-week timepoint, the differences in the SWAL-QOL among the DTD, EA and control groups were not significant (all *P* > 0.05). Such an imbalance between the SSA scores and SWAL-QOL scores at the later stage of the intervention might be due to the distinct emphasis of the two-score assessment systems. As a subjective indicator, the SWAL-QOL scale was specifically designed to evaluate the quality of life of patients from various aspects of physiological, psychological, emotional and social communication [32]. In the later stage after intervention, the enhanced influence from the recovery of other aspects of poststroke may be as confounded factors leading to the weakened intergroup effect in SWAL-QOL scores at 6 weeks after intervention.

To our knowledge, this is the first time that the difference over time in clinical effectiveness was compared among DTD, EA and combination DTD with EA as interventions for PSD.

For the electroacupuncture efficacy shown in our study, most current similar RCTs found the same satisfactory effect on PSD [22]. However, there are currently few RCTs strictly determining the efficacy of DTD for PSD [10]. With low evidence levels and a lack of trend analysis over time, most studies were not comprehensive and were not persuasiveness enough compared with our study.

As the “gold standard” for the evaluation of swallow function, the VFSS was quantified by the VDS in this trial. The decrease in VDS scores (ΔVDS) was regarded as a relatively objective indicator that can reflect the improvement in swallowing function. As mentioned previously, there was a more powerful effect for the combination of DTD with EA than applying it alone. It was necessary to know whether there was a statistical interaction for being responsible for the advantage of DTD + EA. The 2 × 2 factorial design allows researchers to examine the main effects of two interventions simultaneously and explore possible interaction effects [41]. Therefore, we further calculated ΔVDS (between 4 weeks and baseline) and analysed its main effect of group and time, as well as the interaction effect of group and time.

There was a significant main effect of DTD and EA (both *P* < 0.001) but no significant interaction effect between the two (*P* = 0.717). Then, multiple comparisons showed that both DTD and EA had a greater effect on the improvement of VDS compared with the control group, and the joint application of the two interventions achieved more improvement in VDS compared with any single intervention. As there was no interaction according to the data, we seemed to have a certain reason to believe that the obvious advantage of joint interventions came from the simple sum effect of the two intervention measures. A similar phenomenon was observed in SSA scores and SWAL-QOL scores, as discussed above.

It is still difficult to explain the inter mechanism of how acupuncture or Chinese decoction influences swallow function and their biological synergism. According to the present data, it was also difficult to clarify the relationship between the statistical interaction effect and biological synergism, similar to the results of our study. In fact, surely the negative statistical interaction cannot deny the existence of biological synergism taking biological complexity into account [42]. In TCM theory, there are many hypotheses supporting the existence of complex correlations inside the body, which may lead to biological interactions [36].

Traditional Chinese medicine (TCM) theory has attributed PSD to the category of “throat bi”, which is related mainly to meridian obstruction or phlegm dampness, qi deficiency and blood stasis [11, 39]. According to TCM, meridians are the channels through which human tissues communicate with each other and through which Qi and blood run. The meridians are ubiquitous in the body and are an important part of the body's organizational structure [43]. It is difficult to explain Qi from the perspective of modern medicine. As a general interpretation of the human physiological state and pathological evolution, Qi is an intangible and objective substance with certain activity. The body depends on the biochemistry of qi to make all internal tissues sympathetic [44]. In the theory of meridians, the smoothness of meridians and qi plays a vital role in the human body, and it has great significance for the prevention and treatment of stroke [45]. In TCM, “Bi” means obstructed, and “throat bi” means blocked swallowing. “Throat bi” refers to a pathological condition in which swallowing disorders lead to accumulation of food, saliva and sputum in the throat. The symptoms of the disease are in the pharynx and larynx, but the essence is brain damage [46]. TCM believes that the aetiology and pathogenesis of stroke are based on the deficiency of Qi and blood. The disorder of Qi and blood causes cerebral meridians to be blocked or blood to overflow outside the veins [47]. Based on these grounds, the two interventions of this trial affected dysphagia in their respective ways.

Major effector sites of acupuncture for PSD treatment were meridians [48]. Acupuncture has the functions of dredging the meridians, regulating qi, running the blood, regulating the internal organs, clearing the throat, and rejuvenating the mind, all of which achieve an effect on dysphagia [24]. The therapeutic effects of acupuncture treatment occur via needle insertion at specific acupoints on the body surface. Needles are inserted into acupoints via manual rotation to achieve a “de qi” reaction, where the patient perceives an ache or heaviness in the area surrounding the needle [48]. In practice, different acupoint “de qi” reactions have different effects. By electric pulses, EA can directly enhance the effect on the base of mechanical stimulation from the needle [22].

As another aspect of the aetiology and pathogenesis of PSD, phlegm dampness, qi deficiency and blood stasis are considered important targets for DTD therapy [10]. In ancient TCM books, there are some famous prescriptions for the treatment of PSD. Among them, Ditan decoction was commonly used in our department and was good at cleansing phlegm and enlightenment according to long-term clinical observation. Notably, Ditan decoction showed encouraging effectiveness in patients with PSD whose clinical manifestations included excessive saliva, obvious wheezy phlegm in the throat or heavy snoring [10, 49]. As excessive saliva or phlegm in the throat obviously increased the risk of aspiration pneumonia [50], it was reasonable to believe that DTD may decrease the incidence of aspiration pneumonia by cleansing the phlegm or decreasing saliva accumulation in the throat. The effects were verified by the adverse events and complications reported in our study, which showed that both groups treated with the decoction (DTD group and DTD + EA group) had a lower incidence of aspiration pneumonia than the control group (both *P* < 0.05). In addition, DTD contains *Arisaema erubescens* Schott*., Acorus tatarinowii Schott., Poria cocos* Wolf.*, Citrus aurantium* L.*, Pinellia ternata* Breit.*, Citrus reticulata* Blanco.*, Panax ginseng* C. A. Mey.*, Glycyrrhiza uralensis* Fisch.*, and Bambusa tuldoides* Munro. These herbal extracts have been demonstrated to have multiple effects, including replenishing Qi and tonifying the spleen in addition to cleansing the phlegm. The ingredients of DTD were reported to have anti-inflammatory and antioxidant activities [51–53]. Some also possess neuroprotective and anti-stress properties [54–56]. Other studies have shown that herbal medicine, including DTD, alleviates oxidative and inflammatory damage to membrane lipids, nucleic acids, and proteins and promotes neuronal cell survival to maintain their functions [57]. DTD may reduce poststroke symptoms by improving neuroplasticity, reducing the oxidative stress and inflammation induced by stroke, and protecting against neuronal cell death [58]. Therefore, DTD has clinically been used for the treatment of acute cerebrovascular diseases, schizophrenia, neurosis, epilepsy, and poststroke symptoms, including movement, speaking and visual problems [10].

Taken together, DTD and EA might have a certain biological synergism from the action mechanism based on TCM theory considerations. As we have seen in our data, DTD alleviating phlegm or saliva could lead to reduced aspiration and leakage. Meanwhile, the meridians are dredged through acupuncture, which leads to promoting qi and blood by relieving blood stasis. Then, DTD replenishes Qi and tonifies the spleen to increase Qi. Consequently, it is helpful to run Qi and blood, which strengthens resolving phlegm. In turn, resolving phlegm leads to more unobstructed meridians. All those effects derived from joint interventions mutually reinforce, interact with and promote each other, which might be presented as an improvement in swallowing function scores as a manifestation form of biological synergism that demonstrates a more powerful than simple sum effect of a single intervention. This is a preliminary clue for exploring the complex biological synergism between acupuncture and decoction, although it was not consistent with the lack of statistical interaction found in our data of VDS. There were several reasons for this contradiction. First, there were other interferences from other unknown elements that may neutralize the current effect. Second, insufficient statistical efficiency due to a small sample may lead to a false negative result in the statistical interaction. In addition, the VDS indicators were not comprehensive enough for evaluating swallow function to meet the requirements of biological complexity. Further enlarging the sample size, introducing an advanced model and searching more qualified indicators for PSD might be helpful for better understanding the relationship.

Our study also demonstrated sufficient safety for intervention of DTD and EA while ensuring their effectiveness. According to the records, adverse events were all transient and slight. Recently, a meta-analysis showed that DTD administration displayed nonspecific adverse effects, such as drowsiness, sweating, weight gain, constipation, loss of appetite, and dry mouth [10]. In our trial, the patients treated with DTD showed adverse reactions such as nausea or abdominal distension due to taking the decoction too fast. Although acupuncture and EA are widely used in China as complementary or alternative therapies worldwide, few adverse events have been reported. In a meta-analysis of a total of 16 RCTs involving 1,216 patients with PSD, only 16 patients suffered from adverse events related to acupuncture, such as pain and haematoma, but they were all not severe, similar to what appeared in our study [22]. Therefore, we believed that both DTD and EA were safe enough, although they increased the incidence of adverse events compared with the control group (*P* < 0.05).

There are several limitations to the study. First, limited by the requirements of ethics and the specificity in operation, our study was designed as single blind and lacked a sham acupuncture and sham decoction control group, which may lead to an adding bias when analysing. Second, the small sample size of the study impeded stratified analysis of patients according to their different clinical characteristics, which affected the further exploration of available data. In addition, the lack of multicentre involvement and short-term follow-up weakened the persuasiveness of the conclusions.

## 5. Conclusions

Several conclusions can be drawn from our study. First, compared with SRT intervention alone, either adding DTD or adding EA intervention to SRT improved the swallowing function of patients with PSD, which presented an equivalent effectiveness. Second, adding DTD and EA simultaneously to SRT could accelerate and amplify the recovery of swallowing function and correspondingly improve swallowing-related quality of life compared with DTD or EA alone. Nonetheless, there was no statistical interaction effect found on the improvement in PSD according to the data acquired, although the intervention effect of DTD and EA might be synergistic.

In summary, as two important methods of complementary and alternative medicine, both DTD and EA are effective and safe treatment strategies for PSD, which may be applied together or jointly as a powerful supplement to routine treatments. A well-established control trial with a larger sample and longer term is needed to draw more convincing conclusions.

## Figures and Tables

**Table 1:** Baseline characteristics of the subjects (*n* = 76 + 4).

Characteristic	DTD group (*n* = 19 + 1)	EA group (*n* = 20)	DTD + EA group (*n* = 19 + 1)	Control group (*n* = 18 + 2)	*F*/*χ*^2^/*H*	*P*
Median age (IQR) (years)	63.0 (57.3, 73.3)	69.0 (58.5, 76.0)	70.5 (60.0, 78.5)	63.5 (56.3, 74.8)	2.451	0.484
Sex (male/female)	17/3	12/8	15/5	16/4	3.733	0.292
Median course of stroke (IQR) (days)	49.0 (26.0, 54.0)	46.0 (34.8, 51.3)	45.0 (29.0, 53.0)	36.5 (22.3, 54.8)	0.707	0.872
*Comorbidity, n (%)*						
Hypertension	18 (90.0)	17 (85.0)	17 (85.0)	16 (80.0)	0.903^*∗*^	0.971
Diabetes	16 (80.0)	16 (80.0)	15 (75.0)	14 (70.0)	0.829^*∗*^	0.937
Hyperlipidaemia	15 (75.0)	13 (65)	12 (60.0)	11 (55.0)	1.893	0.595
Other chronic diseases	9 (45.0)	6 (30)	7 (35.0)	10 (50.0)	2.083	0.555
aCCI scores	6.85 ± 1.63	7.00 ± 1.49	7.35 ± 1.53	6.30 ± 1.26	1.734	0.167
*Location of lesion, n (%)*						
TACI	7 (35.0)	4 (20.0)	4 (20.0)	2 (10.0)	6.407^*∗*^	0.391
PACI	7 (35.0)	9 (45.0)	9 (45.0)	14 (70.0)		
POCI	6 (30.0)	7 (35.0)	7 (35.0)	4 (20.0)		
NIHSS	9.40 ± 2.50	9.00 ± 3.03	8.00 ± 2.99	8.70 ± 2.08	0.974	0.410

*Note.* ^*∗*^Fisher's exact probability test. Other chronic diseases included mild-moderate heart, liver, and kidney disease. aCCI: age-adjusted Charlson comorbidity index. TACI: total anterior circulation infarct; PACI: partial anterior circulation infarction; POCI: posterior circulation infarction. NIHSS: National Institute of Health Stroke Scale.

**Table 2:** The ER for the treatment of PSD in the four groups (case (%)).

	Completely healed	Markedly effective	Effective	Ineffective	ER (%)
DTD group (*n* = 19 + 1)	0 (0.0)	6 (17.6)	12 (34.3)	2 (20.0)	42.9 ± 14.6^*∗*^
EA group (*n* = 20)	0 (0.0)	11 (32.4)^*∗*^	9 (25.7)	0 (0.0)^*∗*^	48.5 ± 10.8^*∗*^
DTD + EA group (*n* = 19 + 1)	1 (100.0)	16 (47.1)^*∗*^^▲^	2 (5.7)^*∗*^	1 (10.0)	61.2 ± 17.2^*∗*^^▲★^
Control group (*n* = 18 + 2)	0 (0.0)	1 (2.9)	12 (34.3)	7 (70.0)	29.9 ± 13.8
*χ* ^2^/*F*	2.842	25.575	13.562	10.782	16.492
*P*	1.00	<0.01	0.004	0.010	<0.01

*Note*: ^*∗*^compared with the control group, *P* < 0.05; ▲compared with the DTD group, *P* < 0.05; ★compared with the EA group, *P* < 0.05 ER: effective rate.

**Table 3:** Changes in SSA scores after intervention in the four groups over time (means ± SDs).

		Baseline	2 weeks	4 weeks	6 weeks	Simple effect of time	
						*F*	*P*
DTD group (*n* = 19 + 1)		30.25 ± 2.63	28.45 ± 2.28^↑^	25.05 ± 2.11^*∗*^^↑‡^	23.75 ± 2.00^*∗*^^↑‡☨^	31.821	<0.01
EA group (*n* = 20)		29.85 ± 2.93	27.30 ± 2.00^↑^	24.25 ± 1.33^*∗*^^↑‡^	22.75 ± 1.37^*∗*^^↑‡☨^	41.408	<0.01
DTD + EA group (*n* = 19 + 1)		30.50 ± 2.42	26.50 ± 2.35^*∗*^^▲↑^	22.00 ± 2.53^*∗*^^▲★↑‡^	20.40 ± 2.42^*∗*^^▲★↑‡☨^	99.660	<0.01
Control group (*n* = 18 + 2)		29.80 ± 2.44	28.75 ± 2.34^**↑**^	27.40 ± 2.01^**↑‡**^	26.15 ± 2.56^**↑‡☨**^	9.662	<0.01

The simple effect of group	*F*	0.327	4.296	23.781	24.880		
	*P*	0.806	0.007	<0.01	<0.01		

*Notes*: ^*∗*^compared with the control group, *P* < 0.05; ^▲^ compared with the DTD group, *P* < 0.05; ^★^compared with the EA group, *P* < 0.05. ^↑^ compared with baseline, *P* < 0.05; ^‡^ compared with 2 weeks, *P* < 0.05; ^☨^ compared with 4 weeks, *P* < 0.05.

**Table 4:** Changes in SWAL-QOL scores after intervention in the four groups over time (means ± SDs).

		Baseline	2 weeks	4 weeks	6 weeks	Simple effect of time	
						*F*	*P*
DTD group (*n* = 19 + 1)		92.60 ± 9.88	137.60 ± 17.01↑	166.85 ± 17.02^*∗*^^↑‡^	182.70 ± 20.05^↑‡☨^	86.981	<0.01
EA group (*n* = 20)		94.25 ± 12.33	140.50 ± 17.01↑	167.80 ± 13.20^*∗*^^↑‡^	181.25 ± 11.20^↑‡☨^	82.140	<0.01
DTD + EA group (*n* = 19 + 1)		95.15 ± 12.59	156.75 ± 14.26^*∗*^^▲★↑^	182.90 ± 16.95^*∗*^^▲★↑‡^	193.00 ± 19.05^*∗*^^↑‡☨^	114.634	<0.01
Control group (*n* = 18 + 2)		89.45 ± 8.31	126.60 ± 16.79^↑^	147.95 ± 21.81^↑‡^	166.10 ± 26.72^↑‡☨^	64.026	<0.01

The simple effect of group	*F*	1.055	11.669	13.367	6.130		
	*P*	0.373	<0.01	<0.01	<0.01		

Notes: ^*∗*^compared with the control group, *P* < 0.05; ▲compared with the DTD group, *P* < 0.05; ^★^compared with the EA group, *P* < 0.05. ↑ compared with baseline, *P* < 0.05; ^‡^ compared with 2 weeks, *P* < 0.05; ^☨^ compared with 4 weeks, *P* < 0.05.

**Table 5:** Comparisons of ΔVDS among the four groups (means ± SDs).

Groups	*n*	ΔVDS scores
DTD group	19 + 1	33.30 ± 12.40^*∗*^
EA group	20	38.85 ± 12.28^*∗*^
DTD + EA group	19 + 1	47.53 ± 14.80^*∗*^^▲★^
Control group	18 + 2	22.58 ± 10.50

*Note.* ^*∗*^compared with the control group, *P* < 0.05; ^▲^compared with the DTD group, *P* < 0.05; ^★^compared with the EA group, *P* < 0.05.

**Table 6:** Adverse events and complications in the four groups.

Group	*n*	Number of times for intervention	Total number of intervention events (*n *×* *numbers of times)	Adverse events based on total number of intervention events (*n* (%))	New aspiration pneumonia based on number of patients (*n* (%))
DTD group	19	3	57	3 (5.26)	0 (0.00)^*∗*^
EA group	20	3	60	10 (16.67)^*∗*^	3 (15.00)
DTD + EA group	19	3	57	11 (19.30)^*∗*^	0 (0.00)^*∗*^
Control group	18	3	54	0 (0.00)	6 (33.33)
*χ* ^2^	—	—	—	15.088	11.450
*P*	—	—	—	<0.01	<0.01

*Note.* ^*∗*^compared with the control group, *P* < 0.05.

## Data Availability

The data used to support the study are available from the corresponding author Lielie Zhu (e-mail: eillie@126.com).
